# Delayed response task performance as a function of age in cynomolgus monkeys (*Macaca fascicularis*)

**DOI:** 10.1007/s10329-013-0397-8

**Published:** 2013-11-19

**Authors:** H. S. Darusman, J. Call, D. Sajuthi, S. J. Schapiro, A. Gjedde, O. Kalliokoski, J. Hau

**Affiliations:** 1 Department of Experimental Medicine, University of Copenhagen and National University Hospital, Copenhagen, Denmark; 2 Max Planck Institute of Evolutionary Anthropology, Leipzig, Germany; 3 Department of Veterinary Sciences, Michael E. Keeling Center for Comparative Medicine and Research, UT MD Anderson Cancer Center, Bastrop, TX USA; 4 Primate Research Center, Bogor Agricultural University (IPB), Bogor, Indonesia; 5 Department of Neuroscience and Pharmacology, Faculty of Health Science, University of Copenhagen, Copenhagen, Denmark; 6 Center for Functionally Integrative Neuroscience, University of Aarhus, Aarhus, Denmark; 7 Department of Radiology and Radiological Science, Johns Hopkins University, Baltimore, MD USA; 8 Department of Neurology and Neurosurgery, McGill University, Montréal, QC Canada; 9 Department of Anatomy, Physiology and Pharmacology, Faculty of Veterinary Medicine, IPB, Bogor, Indonesia; 10 Blegdamsvej 3B, 2200 Copenhagen N, Denmark

**Keywords:** Working memory, Aging, Non-human primate, Neurodegenerative disease

## Abstract

We compared delayed response task performance in young, middle-aged, and old cynomolgus monkeys using three memory tests that have been used with non-human primates. Eighteen cynomolgus monkeys—6 young (4–9 years), 6 middle-aged (10–19 years), and 6 old (above 20 years)—were tested. In general, the old monkeys scored significantly worse than did the animals in the two other age groups. Longer delays between stimulus presentation and response increased the performance differences between the old and younger monkeys. The old monkeys in particular showed signs of impaired visuo-spatial memory and deteriorated memory consolidation and executive functioning. These results add to the body of evidence supporting the utility of *Macaca fascicularis* in studies of cognition and as a potential translational model for age-associated memory impairment/dementia-related disorders.

## Introduction

Aged monkeys display many of the key cognitive deficits associated with human aging and dementias. Ample evidence of the utility of aged non-human primates (NHP) as models for human age-related neurodegenerative diseases has been provided (Voytko 1996; Duan et al. 2003; Voytko and Tinkler 2004; Bartus and Dean 2009; Nagahara et al. 2010). Several behavioral tasks for assessing various cognitive domains that were developed in research into human neuropsychology have been successfully adapted for use with NHP. These include delayed response tasks (Kojima 1980; Kojima and Goldman-Rakic 1982; Tomasello and Call 1997), where delays of various lengths are imposed between the presentation of a stimulus and the expected response (Voytko 2000; Rodriguez and Paule 2009; Nagahara et al. 2010).

The present investigation focused on the decline of working memory and long-term (up to 24-h) memory as a function of age in aging cynomolgus monkeys (*Macaca fascicularis*). Anecdotal evidence from caregivers suggests that old cynomolgus monkeys have greater difficulty in recalling locations—for instance, retrieving previously hidden food—than do younger macaques. Both aged rhesus monkeys and cynomolgus monkeys have been demonstrated to exhibit brain lesions similar to those of humans suffering from neurodegenerative diseases such as Alzheimer’s disease (Sloane et al. 1997; Oikawa et al. 2010; Kimura et al. 2003; Nakamura et al. 1998). To systematically test the hypothesis that spatial and working memory are impaired in aged cynomolgus monkeys, we adopted three memory tasks that were previously successfully employed in studies of other non-human primate species (Amici et al. 2010; Barth and Call 2006; Call 2001; Beran et al. 2005; Martin-Odas and Call 2011): the short-term memory test (STMT), the long-term memory test (LTMT), and the memory load test (MLT). These tests are relevant in assessing dementias, since they assess the functioning of the prefrontal cortex (Funahashi et al. 1993; Funahashi 2006) and the hippocampal region of the brain, which is strongly affected by aging (Wu et al. 2008) and Alzheimer’s disease (Rodriguez and Paule 2009).

The STMT consists of assessing each subject’s memory of the location of an object. A food reward (bait) is hidden in one of three identical, opaque containers (cups) which are then presented to the subject after a specified delay. The STMT is easy to conduct and has previously been successfully used to test spatial working memory in monkey species, including the spider monkey (*Ateles geoffroyi*), the capuchin monkey (*Cebus apella*), and the cynomolgus monkey (Amici et al. 2010). Typically, individuals show higher retrieval accuracies with short delays than with long delays (Tomasello and Call 1997; Amici et al. 2010). Here, we used 30-, 60-, and 120-s delays.

The LTMT has been used to test long-term memory and memory consolidation in several great ape species (Martin-Odas and Call 2011). The LTMT is similar to the STMT, with bait hidden in one of three opaque cups. However, in the LTMT, each cup is unique (differing in shape and color), and the delays between the baiting of the cups and the attempted retrieval of the bait are considerably longer than in the STMT. Since the cups differ in their external features, subjects may be able to use this visual information along with or instead of spatial information only to retrieve the bait. Retrieval accuracy in great apes has been reported to follow a U-shaped curve, with peak performance occurring at no delay and 24 h after the baiting event took place, suggesting that the LTMT revealed a possible memory consolidation process (Martin-Odas and Call 2011).

The MLT tests spatial working memory: to succeed, subjects are required to remember the location of two baits hidden in two of six identical opaque cups arranged in a straight line. The subjects are tested immediately, or following a 30-s delay. The MLT is also easy to conduct and has been used to assess spatial memory in apes (Call 2001; Beran et al. 2005; Hribar and Call 2011). Typically, apes show higher retrieval accuracies when the baited cups are adjacent to one another compared to when they are not (i.e., when there is at least one empty cup between the baited cups). Additionally, apes show higher retrieval accuracy when cups at the ends of the row are baited compared to when baited cups are located between two empty cups (Hribar and Call 2011).

Impaired working and spatial memory should translate into poor performance in the STMT, LTMT, and MLT tests. As memory declines with age, we hypothesized that performance in these tests would deteriorate from young to older cynomolgus monkeys. Moreover, this trend was expected to be particularly pronounced in tasks that are more difficult in terms of delay or number of cups available.

## Methods

### Subjects

Eighteen adult cynomolgus monkeys raised in colony cages were grouped by age, adapting the age classifications of Moss et al. (2007) from humans to macaques. Macaques between 4 and 9 years of age were categorized as “young” (*n* = 6, 3 males and 3 females), and those between 15 and 16 years of age were considered to be “middle-aged” (*n* = 6; 3 males and 3 females). Six macaques (3 males and 3 females) that were 20 years of age or older were categorized as “old.” Subject age was determined from birth certificates for the animals born in captivity, and from dental scaling (Swindler 2002) for animals born in the wild. The subjects’ characteristics are described in Table 1. All subjects were clinically healthy and tested monthly to confirm that they were tuberculosis-free. Potential gender-related differences were not investigated due to the small number of animals of each sex in each age group.

**Table 1:** Characteristics of the subjects

Identity (tattoo)	Age group	Sex	Body weight (kg)	Dental scale (age group)
FA9103	Young	Female	2.6	M2/M(3) (4–9 years old)
C2538	Young	Female	2.6	M2/M(3) (4–9 years old)
C0032	Young	Female	3.0	M2/M(3) (4–9 years old)
C0744	Young	Male	4.3	M2/M(3) (4–9 years old)
C3852	Young	Male	4.0	M2/M2 (4–6 years old)
C2480	Young	Male	5.0	M2/M(3) (4–9 years old)
T3615	Middle-aged	Female	2.7	M2/M3 (10–19 years old)
T3619	Middle-aged	Female	3.1	M2/M3 (10–19 years old)
FC9095	Middle-aged	Female	3.7	M3/M3 (10–19 years old)
T3051	Middle-aged	Male	5.2	M3/M3 (10–19 years old)
T2895	Middle-aged	Male	5.6	M3/M3 (10–19 years old)
K30	Middle-aged	Male	5.6	M3/M3 (10–19 years old)
I1166	Old	Female	2.9	M3/M3-H (>20 years old)
I1112	Old	Female	2.9	M3/M3-H (>20 years old)
C5545	Old	Female	4.0	M3/M3-H (>20 years old)
T3311	Old	Male	7.0	M3/M3-H (>20 years old)
T3296	Old	Male	5.2	M3/M3-H (>20 years old)
C2466	Old	Male	5.1	M3/M3-H (>20 years old)

Prior to beginning data collection, all subjects were housed at the AAALAC-accredited Primate Research Center IPB (Bogor, Indonesia) in pairs or social groups of various sizes, with access to indoor and outdoor areas. During testing, subjects were housed indoors in adjacent individual cages, which permitted restricted tactile contact. The adjacent cages consisted of two joined individual cages—approximately 150 × 75 × 50 cm (W × L × H)—that were separated by a perforated acrylic glass window allowing adjacent monkeys to see one another and to engage in protected tactile contact. Tests were conducted over a 3-month period (August–October 2011), following a 1-month period of acclimatization to the housing environment. Subject housing conditions (before, during, and after the experiment) and the test procedures were approved by the Primate Research Center IPB’s Animal Care and Use Committee (ACUC).

### General experimental procedures

Macaques were fed fruits and a standard monkey chow diet (Harlan^®^ 2050 Teklad Global 20 % Protein Primate Diet, Indianapolis, IA, USA) twice a day. Tap water was available ad libitum. The subjects were habituated to the procedures and the experimenter, and they voluntarily sat down and faced the experimenter once the test stimuli were prepared. The basic procedure was the same for all tests. Although we attempted to administer the same number of tests and trials to all monkeys, some subjects became too aggressive or too fearful during testing and were unable to complete the whole set of trials. In such cases, we discontinued testing and used the portion of the data that was available until then, as specified below.

The experimenter and the subject were located approximately 50 cm apart, separated by the bars of the cage front. Facing the subject, the experimenter prepared the test stimuli (1 or 2 pieces of fruit, depending on the test) on a tray resting on an L-shaped steel support attached to the outside of the subject’s cage. At the beginning of each trial, the tray was out of the subject’s reach. The experimenter showed the subject that all cups were empty by letting the cups rest on their side with the open top of the cup directed toward the subject. In full view of the subject, the cup(s) were then baited according to a randomized schedule, hiding the food reward. The delays were measured from the time that the bait was hidden and the cups were properly aligned until the tray and cups were moved within reach of the subject.

The experimenter wore dark sunglasses in order to eliminate potential inadvertent cuing from eye movements. In the selection phase, the subjects were allowed to choose by touching a cup (two cups in the case of the MLT). If a baited cup was selected, the subject received the bait as a reward. In the case of an incorrect choice, the experimenter simply showed that the selected cup was empty and withdrew the tray. The bait was a piece of either sliced apple or guava approximately 3 × 4 × 2 cm (W × L × H) in size.

The subjects were habituated to the test apparatus and the procedure in three steps. First, the bait was presented on the tray and the subject was allowed to retrieve it. If the subject retrieved the bait, the habituation progressed to the second stage, in which the bait was presented partially hidden under a cup on the tray. If the subject retrieved the partially hidden bait, it advanced to the third step, which consisted of presenting the bait completely hidden under a single cup. Once the subject was able to retrieve this bait, the subject was deemed ready for testing. During baiting, the experimenter made sure that the subjects were facing the tray and were watching as the bait was placed under one of the cups. After the baiting took place, and prior to the subjects’ choice, the cups remained in full view of the subjects during the various delays (see below).

Ideally, to prevent a non-mnemonic problem-solving strategy, such as waiting near to or staring at the baited cup during the delay, the cups can be hidden behind an occluder after completing the baiting. However, we were unable to do this because the placement and removal of the occluder disturbed subjects in preliminary trials to the point that they refused to participate. Therefore, we decided to leave the cups in full view of the subject during the delay. Since subjects did not wait near to or stare at the cups throughout the delay, it seems unlikely that such non-mnemonic strategies can fully explain our results, although other such strategies (e.g., positioning the body in front of the correct stimulus) could have contributed. Our use of several delay lengths and the corresponding results also militate against non-mnemonic strategies as the sole explanation for our results. All subjects received the test in the same order, which was STMT, LTMT, and MLT.

### Specific experimental procedures

#### Short-term memory test (STMT)

For the STMT, three identical opaque plastic cups (10 cm in diameter) were arranged in a line and spaced 5 cm apart on a 45 × 30 cm plastic tray. The STMT consisted of a series of trials in which four delays—0 (no delay), 30, 60, and 120 s—were combined with all three bait positions. In what was termed a “module,” all 12 possible “delay × position” combinations were used. In order to prevent the subject from learning the sequence of the trials, four randomized modules were used. Each subject received a total of 48 trials (4 modules of 12 trials each) except for 6 monkeys (2 in each age group), who received a total of 36 trials (3 modules of 12 trials each). Only the cup touched first was recorded for each trial.

#### Long-term memory test (LTMT)

For the LTMT, three opaque cups differing in shape and color but not volume were used (approximately 9–10 cm in diameter and 7–8 cm in height). The cups were lined up on a 55 × 20 cm tray and spaced 9–10 cm apart. The delays utilized for the LTMT were 0 (no delay), 2, 4, 8, 12, and 24 h. Each subject was presented with only one module consisting of all 18 possible “delay × position” trial combinations, counted from 6 delays × 3 cup positions (right, middle, and left). Only the cup touched first was recorded.

#### Memory load test (MLT)

Six identical opaque cups (7 cm in diameter) were lined up on a 55 × 20 cm tray. Two cups were baited (15 possible bait combinations) and 2 delays were used—0 (no delay) and 30 s, producing a module of 30 possible trial combinations. Seven subjects took part in a total of 120 trials (4 modules of 30 trials each) and 5 others (2 young, 1 middle-aged, and 2 old) took part in a total of 90 trials (3 modules of 30 trials each). The two cups that were touched first were recorded for each trial. The subject received the reward from the first baited cup touched before it touched the second one.

### Scoring and data analysis

The primary dependent variable for the tests was retrieval accuracy or percent correct, defined as the percentage of trials in which subjects chose the cup(s) that contained the bait. In addition to overall correct choices (the first two cups touched), the MLT test also yielded “half correct” responses, in which the subject correctly chose only one of the two baited cups. In every test, subjects in some of the trials did not select any cup. If the subject did not respond in a trial with no delay, the subject was deemed to be unmotivated and testing was stopped, to be resumed at a later time. If a motivated subject failed to respond in a trial with a delay, we analyzed the data in two different ways. In the main analysis, we dropped the trials with no response from the analyses, and in a subsequent analysis we considered those trials to be incorrect because they could be interpreted as a possible failure in memory recall—the subject may have forgotten the existence of the hidden bait.

To calculate inter-observer reliability, we videotaped 70, 57, and 90 additional trials in the STMT, LTMT, and MLT tasks, respectively, and the first author and JC scored them independently. Interobserver reliability was excellent in all cases (Cohen’s kappas: STMT = 0.90; LTMT = 0.83; MLT = 0.97). The effect of delay and age on the percent of correct responses was analyzed in all experiments. Additionally, we estimated the percent of correct responses in the LTMT as a function of delay using a best-fit second-order polynomial, and analyzed the effect of cup position and inter-cup distance on the percent of correct responses in the MLT. Since the raw data from the STMT and LTMT violated the assumptions of normality and homogeneity of variance, nonparametric statistics were used to analyze the effects of delay and age on spatial memory. All other tests were conducted on normally distributed data. We used two-tailed *p*-values (*p* < 0.05 was considered significant) in all statistical tests, except when we analyzed the relation between delay and retrieval accuracy in the LTMT, since Martin-Odas and Call (2011) found a quadratic relation between these two variables.

## Results

### Short-term memory test (STMT)

**Fig. 1:**
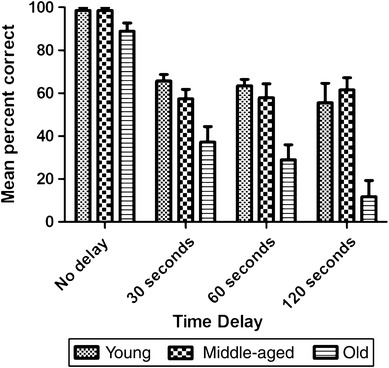
Mean percent of correct trials in the short-term memory test (STMT) as a function of age group and delay. Each data value is presented as a percent of the total number of trials. *Error bars* represent ± standard error of the mean (SEM)

Subjects showed significantly greater retrieval accuracy after shorter delays (Friedman test: *χ*_3_^2^ = 37.66, *p* < 0.001, *N* = 18). Moreover, the old group displayed the worst spatial memory of the three age groups (Kruskal–Wallis test: *χ*_2_^2^ = 11.51, *p* < 0.01, *N* = 18). Overall, we found significant differences between groups (Fig. 1) for each of the delays (Kruskal–Wallis tests: 0 s: *χ*_2_^2^ = 6.04, *p* < 0.05; 30 s: *χ*_2_^2^ = 9.89, *p* < 0.01; 60 s: *χ*_2_^2^ = 9.83, *p* < 0.01; 120 s: *χ*_2_^2^ = 10.62, *p* < 0.01). Mann–Whitney post hoc exact tests revealed significantly poorer retrieval accuracy by the old subjects compared to the other two age groups in the 30-s (young: *p* < 0.01, middle-aged: *p* < 0.05), 60-s, and 120-s (young: *p* < 0.01, middle-aged: *p* < 0.01) trials. In contrast, the differences between groups were not significant at a delay of 0 s (*p* > 0.09 in all cases).

Only old subjects failed to respond in 15 of the 264 trials (5.68 %), mostly in the 120-s delay condition (13 out of 66 trials). A re-analysis after scoring those trials as errors did not alter the results reported above. In particular, both delay (Friedman test: *χ*_3_^2^ = 37.66, *p* < 0.001, *N* = 18) and age (Kruskal–Wallis test: *χ*_2_^2^ = 11.51, *p* < 0.01, *N* = 18) significantly affected performance.

### Long-term memory test (LTMT)

**Fig. 2:**
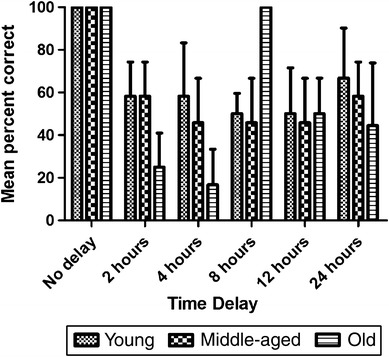
Mean percent of correct trials in the long-term memory test (LTMT) as a function of age group and delay. Each data value is presented as a percent of the total number of trials. *Error bars* represent ± standard error of the mean (SEM)

As with the STMT, retrieval accuracy varied with delay (Friedman test: *χ*_5_^2^ = 16.49, *p* = 0.006, *N* = 12). In contrast, there were no significant differences between the age groups (Fig. 2; Kruskal–Wallis test: *χ*_2_^2^ = 0.26, *p* = 0.88, *N* = 12). In the no-delay trials, all subjects responded correctly in all trials. Young and middle-aged subjects performed better than chance in all trials, while the old subjects fell below chance for the 2- and 4-h delays and, unexpectedly, they scored perfectly at the 8-h delay. The relationship between retrieval accuracy and delay appeared to follow a quadratic function (Fig. 2). However, this could not be statistically confirmed for either the whole sample or for each age group separately (young: *F*_3_ = 3.40, *p* > 0.15; middle-aged: *F*_3_ = 3.01, *p* > 0.18; old: *F*_3_ = 0.03, *p* > 0.95).

Subjects failed to respond in 35 of the 216 trials (16.2 %), with old subjects accounting for 27 of these 35 trials. However, a re-analysis of the data after scoring no-response trials as errors left the results reported above largely unchanged. In particular, delay (Friedman test: *χ*_3_^2^ = 28.35, *p* < 0.001, *N* = 12) but not the age of the subjects (Kruskal–Wallis test: *χ*_2_^2^ = 1.43, *p* = 0.49, *N* = 12) significantly affected performance. The only variation compared to the previous results was that the quadratic function between delay and retrieval accuracy became significant for young individuals (*R* ^2^ = 0.83, *F*_3_ = 7.11, *p* < 0.05 one-tailed), but it remained nonsignificant for the other two age groups (middle-aged: *R* ^2^ = 0.70, *F*_3_ = 3.56, *p* = 0.081 one-tailed; old: *R* ^2^ = 0.39, *F*_3_ = 0.74, *p* = 0.27 one-tailed). The lowest retrieval accuracy occurred 12 h after the baiting. In fact, the retrieval accuracy for young and middle-aged monkeys at 12 h was significantly lower than their response accuracy after both no delay (Wilcoxon test: *T* = 28, *p* < 0.01, *N* = 7, one-tailed) and a 24-h delay (Wilcoxon test: *T* = 15; *p* < 0.05, *N* = 5, one-tailed).

### Memory load test (MLT)

**Fig. 3:**
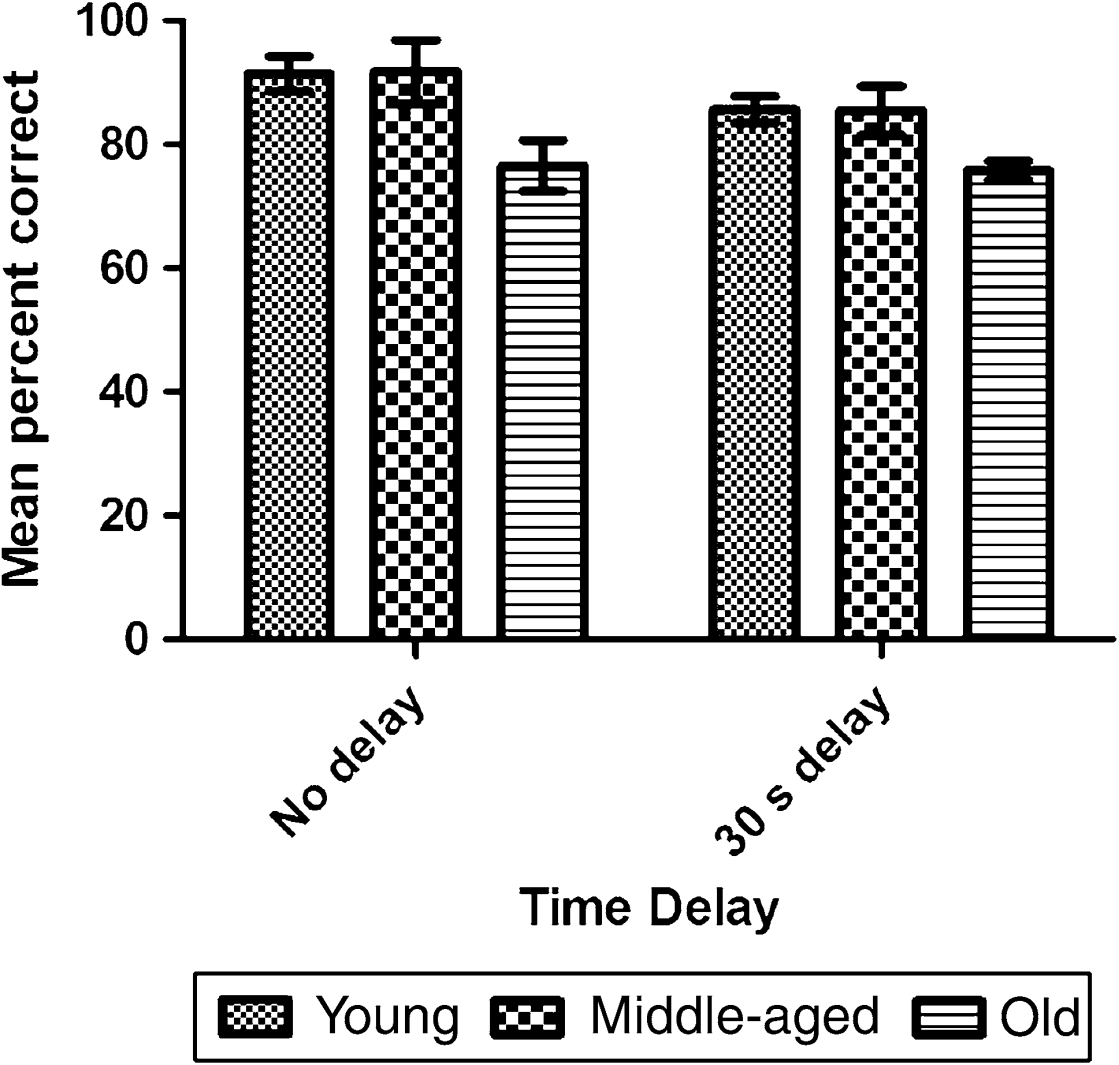
Mean number of correct responses in the memory load test (MLT) as a function of age group and time delay. Each data value is presented as the average number of baits retrieved per trial. *Error bars* represent ± standard error of the mean (SEM)

A mixed-model ANOVA on the number of correct choices as a function of age and delay revealed a significantly higher retrieval accuracy in the 0-s delay compared to the 30-s delay (Fig. 3; *F*_1,9_ = 5.25, *p* = 0.048). There was also a significant effect of age (*F*_2,9_ = 5.36, *p* = 0.029) but no significant delay × age interaction (*F*_2,9_ = 0.87, *p* = 0.45). Post-hoc LSD tests indicated that old subjects performed significantly worse than young (*p* = 0.02) and middle-aged subjects (*p* = 0.019).

**Fig. 4:**
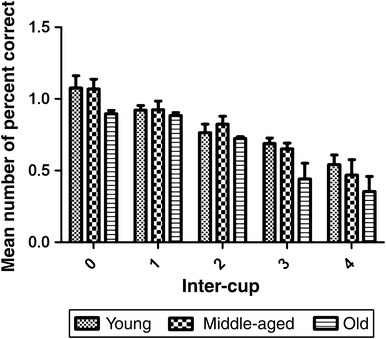
Mean number of correct responses in the memory load test (MLT) as a function of age and inter-cup distance (the number of cups separating the baits). Each data value is presented as the average number of baits retrieved per trial. *Error bars* represent ± standard error of the mean (SEM). The inter-cup distance refers to the number of empty cups between the two baited cups

Out of all the pairs of responses recorded (recall that each trial required two responses), monkeys selected two adjacent cups in 79.2 % of the trials. A mixed-model ANOVA on the percentage of trials in which subjects selected adjacent cups as a function of age and delay revealed a significant effect of age (*F*_2,9_ = 6.73, *p* = 0.016) but not of delay (*F*_1,9_ = 4.39, *p* = 0.066) or delay × age (*F*_2,9_ = 0.06, *p* = 0.94). Post-hoc LSD tests indicated that old subjects performed significantly worse than young (*p* = 0.025) and middle-aged subjects (*p* = 0.007). The disproportionate number of responses to adjacent cups (only 33 % of the trials featured adjacently placed baits) resulted in all age groups being significantly better at retrieving both baits when they were placed in adjacent, as opposed to non-adjacent, cups. In fact, subjects’ retrieval accuracy significantly decreased in inverse proportion to the distance between the baited cups (Fig. 4; *F*_4,36_ = 37.59, *p* < 0.001), independently of age (*F*_8,36_ = 0.62, *p* = 0.75). Nevertheless, age differences were evident overall (*F*_2,9_ = 5.32, *p* = 0.03) and post hoc LSD tests indicated that old subjects performed significantly worse than young (*p* = 0.017) and middle-aged subjects (*p* = 0.024).

Re-analyzing the whole data set (not just those responses to adjacent cups) after re-scoring as incorrect those trials in which subjects failed to respond in one or two of their choices (out of the possible 2580 responses, subjects failed to respond 51 times) still revealed a significant effect of age (*F*_2,9_ = 7.82, *p* < 0.05) but not delay (*F*_1,9_ = 2.99, *p* = 0.118) or delay × age (*F*_2,9_ = 0.57, *p* = 0.59). Post-hoc LSD tests indicated that old subjects still showed a significantly worse retrieval accuracy than both young and middle-aged subjects (*p* = 0.008). We did not conduct this analysis on adjacent responses because we could not assign a value to trials without responses.

## Discussion

Old cynomolgus monkeys displayed poorer retrieval accuracy than did their young and middle-aged counterparts in several delayed response tasks. In general, the longer the delay between baiting and retrieval, the worse the monkeys’ performance became. The only possible exceptions to this were found when the delay was too short or too long, in which case performance remained above chance, particularly for young and middle-aged monkeys.

In STMT, the old subjects’ performance was inversely correlated with the length of the delays, falling below the chance level in conditions with long delays. This was not due to errors of omission, because even after removing these from the data, performance remained below chance. Similar to the current study, Amici et al. (2010) found that adult cynomolgus monkeys performed above chance after a short delay between baiting and retrieval, but that their performance deteriorated with longer delays. In contrast, spider monkeys performed above chance on all delays. One possible explanation for this difference may be related to the different daily dispersal patterns of cynomolgus and spider monkeys. It has been hypothesized that species with a high level of fission–fusion dynamics, such as great apes and spider monkeys, have relatively enhanced cognitive skills, such as inhibitory control, memory, and analogical reasoning (Barrett et al. 2003; Aureli et al. 2008).

Old monkeys also occasionally failed to respond in some trials, particularly those with longer time delays. Young and middle-aged individuals also occasionally failed to respond in some trials, but less frequently. Old subjects may become more frustrated than monkeys of the other age groups when long delays are implemented, and thus unmotivated to perform under these conditions. However, the lack of a significant difference between old monkeys and the other two groups in the no-delay conditions in the STMT and LTMT suggests that other differences were unlikely to be due to motivational differences between the groups. Interestingly, a difference between old monkeys and the others appeared in the no-delay condition of the MLT. The additional memory load required to keep two baited locations in memory (out of six possible ones) may explain this apparent discrepancy between tasks.

Thus, our data suggest that old monkeys were as eager as other age groups to retrieve the baits, but had greater difficulty doing so, especially under more cognitively demanding conditions. It is conceivable that older monkeys had forgotten about the bait after long delays. However, the relatively small percentage of errors by omission paired with the small number of subjects prevented us from systematically analyzing this. Future studies should address these issues.

The LTMT, as well as other tests related to delayed response, engages several cognitive faculties, including inhibition control, memory encoding, and memory retrieval (Amici et al. 2010). Together with the STMT, the LTMT ostensibly functions as a spatial memory assessment, but the use of cups with unique visual features enables subjects to solve this task using nonspatial visual cues. Although rhesus monkeys perform well on visual memory, and even show some qualitative similarities with humans (Elmore et al. 2011), long delays may require other memory systems such as working memory which are regarded as more robust and relevant for long-term memory (Scott et al. 2012). It remains unclear whether subjects focused on the cups’ features, their spatial location, or a combination of the two. This is another question that should be addressed in future studies.

Our findings, along with our small sample size of visuospatial memory task trials, agree with an earlier study by Anderson et al. (1993), which revealed that old macaques committed more errors in visuospatial memory tasks and presented greater behavioral rigidity compared with the young ones. Another study of the same species by Anderson et al. (1996) reported that old macaques required more trials to learn a simple visual discrimination.

The LTMT was primarily aimed at assessing memory processing and consolidation. Long delays between food baiting and retrieval substantially affected the performance of all subjects, not just the oldest ones, who fared particularly poorly in the 2–4-h delay conditions. There was some evidence, albeit weak, of a consolidation function in the young monkeys so that their retrieval accuracy gradually decreased over time to a low point and then returned to the levels observed 24 h earlier. Martin-Odas and Call (2011) found a similar U-shaped retrieval performance curve in great apes. We consider these observations to be related to the time required for memory processing, and possibly other factors, such as sleep. Memories may not be lost with the intermediate delays, but rather become temporarily inaccessible.

In the old group, the quadratic trend was not significant, suggesting that the memory of the bait placement may not have been consolidated (Martin-Odas and Call 2011). Since the middle-aged subjects also showed no significant return in performance over time, it is conceivable that the impaired memory consolidation suggested by the LTMT may be an early indicator of age-associated memory problems in cynomolgus monkeys. These results agree with those from rhesus monkeys (Rodriguez and Paule 2009; Scott et al. 2012), where delays possibly shift the visual short term memory to active working memory. In our study, old subjects performed far below chance after an initial delay, suggesting that working memory might be diminished and unable to show a significant recovery, in contrast with young and middle-aged subjects. This suggests a possible unsuccessful shift from visual short-term to active working memory and a memory consolidation process.

An effect of age was also evident in the MLT, again with older monkeys finding less food than their young or middle-aged counterparts. Delay also played a role, but only when trials without responses were excluded from the analysis. In contrast to the weak effect of delay, the bait positions strongly influenced performance in this test. In this task, all subjects were more successful at retrieving food when it was placed under two adjacent cups. Moreover, the further apart the baited cups, the more difficult the task became. A similar difficulty in retrieving non-adjacent baited cups in great apes has been suggested to be an effect of poor inhibitory control (Call 2001) or poor memory encoding (Beran et al. 2005).

The old subjects were the worst at retrieving non-adjacent baits, indicating an age-related impairment of inhibitory control or memory encoding/retrieval issues. The nature of the cognitive decline in the old subjects in the present study is unclear. Degeneration of the prefrontal cortex and hippocampus has been identified as a cause of the delayed response performance deficit in aged rhesus monkeys (Wu et al. 2008). Whereas it is well recognized that spatial memory is highly affected by aging in NHP (Wang et al. 2011; Foster et al. 2012), the loss of episodic memory integrity and impaired executive function—both of which were arguably exhibited by the old subjects—are hallmarks of Alzheimer’s disease and related dementias (Blennow et al. 2006; Perrin et al. 2009; Salmon and Bondi 2009; Gleichgerrcht et al. 2010). Beta-amyloid-positive senile plaques have been detected in aged cynomolgus monkeys (Nakamura et al. 1998; Kimura et al. 2003), and there is some evidence of tau protein accumulation (Oikawa et al. 2010). Although in rhesus monkeys the relation between the amyloid burden and dementia-related symptoms of Alzheimer’s disease is debatable (Sloane et al. 1997; Buccafusco 2008), the drastic reduction in performance seen in old subjects in the present study may, in part, be of a pathological nature.

Despite the possibility of changes in brain anatomy and function, differences in cognitive performance among age groups could also be influenced by differences in endocrine hormone levels, as described by Lacreuse and Herndon (2009). In humans, NHPs, and rats, both estrogen and androgen receptors are present in several areas associated with cognitive function, especially the hippocampus, and modulate certain functions in relation to cognitive function, such as increasing cerebral blood perfusion, influencing neuronal connectivity in hippocampus, and neuroprotective effects (Voytko et al. 2009). Older individuals tend to have lower levels of both sex steroid hormones; therefore, lower hormone levels in old subjects may contribute to poorer spatial memory performance compared with young and middle-aged subjects.

In conclusion, aged cynomolgus monkeys displayed impaired spatial memory compared to younger conspecifics, as evidenced by their lower performance in a series of three delayed response tasks. Specifically, old subjects showed signs of diminished visual-spatial working memory, impaired memory consolidation, and possibly poorer inhibition control/executive functioning. Our results may add to the body of evidence (Voytko and Tinkler 2004) which has identified similarities in the cognitive profiles of nonhuman primate models of aging, Alzheimer’s disease, and menopause with their human counterparts. The study determined the neural substrates of aging-related cognitive dysfunction, known as the cholinergic hypothesis of memory dysfunction in NHP models. Although the underlying (neurological) causes remain to be elucidated, aged cynomolgus monkeys mirror many of the cognitive deficits seen in aged humans, and thus show promise as a translational model for human age-associated memory impairments/dementias.
